# Chemicals of concern in select packaged hair relaxers available on the Kenyan market: an examination of ingredient labels and measurement of pH

**DOI:** 10.3389/fpubh.2025.1532113

**Published:** 2025-04-16

**Authors:** Beatrice N. Irungu, Adana A. M. Llanos, Mary Nyangi, Teresa Olisa, Esther Matu, Amber Rockson, Alexis Schaefer, Adiba Ashrafi, Mary Beth Terry, Jasmine A. McDonald, Janet Nudelman, Laura Dobbs Gillan, Pujeeta Chowdhary, Sabina Wachira, Cecilia Kimani

**Affiliations:** ^1^Center for Traditional Medicine and Drug Research, Kenya Medical Research Institute, Nairobi, Kenya; ^2^Department of Epidemiology, Mailman School of Public Health, Columbia University Irving Medical Center, New York, NY, United States; ^3^Herbert Irving Comprehensive Cancer Center, Columbia University Irving Medical Center, New York, NY, United States; ^4^African-Caribbean Cancer Consortium (AC3), Philadelphia, PA, United States; ^5^Center for Community Driven Research, Kenya Medical Research Institute, Kirinyaga, Kenya; ^6^Campaign for Safe Cosmetics, Breast Cancer Prevention Partners, San Francisco, CA, United States

**Keywords:** Hair products, personal care products, hair relaxers, cosmetics, chemicals of concern, pH, ingredients labels

## Abstract

**Background:**

There is an emerging interest in the investigation of hair relaxers as important sources of exposure to chemicals of concern (CoCs) and their associated adverse health effects. We focused on documentation of CoCs by examining labels of selected relaxers currently available on the market in Nakuru and Embu Counties, Kenya and measured the pH profiles to ensure compliance with Kenya Bureau of Standards.

**Methods:**

We enrolled 746 women aged 15–50 years in a cross-sectional study, which ascertained participants’ sociodemographic characteristics, personal care products use in the last 7–14 days and ever use of hair dyes and chemical relaxers including the brand names of products used. Based on participants’ questionnaire responses and product availability at beauty shops and supermarkets, we purchased 22 different relaxer products. The label of each product was reviewed and we recorded relaxer strength, manufacturer and location, listed ingredients, and other claims. To identify CoCs, we cross-checked the list of ingredients against the Campaign for Safe Cosmetics’ (CSC) Red List and European Union’s prohibited and restricted substances (Annex II and III respectively), Regulation 1223/2009 on cosmetics. The pH profiles of each product were determined using a benchtop pH meter.

**Results:**

Twenty-seven CoCs were documented upon examination with each relaxer listing more than one CoC. Thirteen out of 27 (48.2%) were fragrance chemicals with d-limonene/limonene and linalool, each being listed as an ingredient in 9 products. Fourteen (63.6%) relaxers had undisclosed ingredients listed as ‘fragrance’ and/or ‘parfum’. Six of the identified CoCs are classified as Tier 1 (Do not use for everyone) per CSC Red List while 14.8% (4) are prohibited and 55.6% (15) are restricted substances per EU regulations. The pH values of the relaxers were within Kenya Bureau of Standards required range of 11–13.

**Conclusion:**

These findings create awareness of CoCs listed on labels of selected hair relaxers. This justifies the need for consumer education on potentially harmful chemicals and their associated risks. Further, our findings justify the need for laboratory study to evaluate and quantify CoCs that are listed as well as those that are not listed on the label.

## 1 Introduction

The marketing and advertising of hair care, beauty, and cosmetics products often reinforce Eurocentric beauty standards, including, long, straight hair and lighter skin tones. These standards predominantly target femme-identifying individuals and shape societal perceptions of beauty, influencing the consumption of specific products, such as chemical hair relaxers, among Black and African ancestry women and other women of color (1, 2). The widespread use of these products across the African Diaspora is well-documented. For instance, the prevalence of hair relaxer use among Black American women and women in West Africa has been estimated at approximately 90% (3, 4), with initiation often occurring early in life (5, 6).

In Africa, the use of hair relaxers and chemical straighteners is common. A study by Khumalo et al. in Langa Township, Cape Town, South Africa, reported that 78% of schoolgirls (ages 6–17) and 49.2% of women (ages 18–86) with afro-textured hair used chemical relaxers (7). Similarly, Etemesi examined relaxer use among women aged 15–51 years in Nakuru County, Kenya, finding that 59% reported ever using relaxers, while 41% reported continued, long-term use despite recognized risks such as burns and hair loss, as well as potential unknown hazards (8).

Hair relaxers are classified as either “lye” or “no-lye” based on the active alkaline agent responsible for chemically straightening the hair. Lye relaxers contain sodium hydroxide, whereas no-lye relaxers use either calcium, lithium, or potassium hydroxide, or guanidine carbonate. These products function by breaking disulfide bonds in the hair’s protein structure, thereby loosening its natural curl pattern (9). In addition to their active alkali components, hair relaxers also contain other ingredients such as preservatives and fragrances, some of which have been identified as chemicals of concern (CoCs) due to their association with various adverse health effects (10–12). For example, endocrine-disrupting chemicals (EDCs), including nonylphenols, parabens, and phthalates, are present in some relaxers and have been linked to an increased risk of breast cancer and other endocrine-mediated health outcomes (13). Exposure to CoCs in relaxers occurs through dermal absorption, inadvertent ingestion, and potential contamination of the indoor environment (14).

Empirical evidence supports the association between chemical hair product use and increased breast cancer risk (3, 4, 15, 16). In the Women’s Circle of Health Study, Llanos et al. (3) reported that White women who used both hair relaxers and hair dyes had an elevated risk of breast cancer. Similarly, findings from Eberle et al. (15) in the Sister Study indicated a higher risk of breast cancer among Black women who reported using hair straightening products and permanent hair dyes. Brinton et al. (4) in the Ghana Breast Health Study, observed a significant association between long-term use of chemical relaxers particularly no-lye formulations and increased breast cancer risk. Additional adverse health outcomes linked to hair relaxer use include earlier onset of menarche (6, 17), alterations in estrogen metabolism (18), reduced fertility (19), and elevated risks of uterine fibroids (20), uterine cancer (21, 22), and ovarian cancer (23).

Prior studies have documented the presence of CoCs on the labels of personal care products (PCPs). For example, Johnson et al. examined labels of 546 PCPs in California, United States, and found that 65% listed CoCs as ingredients (14). Similarly, a study conducted in Curitiba, the capital of a southern Brazilian state, by Uber et al. reported that 295 of 398 children’s cosmetics analyzed contained ‘parfum,’ a known CoC, as an ingredient (24).

The beauty industry in Kenya has expanded substantially, with an estimated value exceeding Ksh 20 billion as of 2023 (25). Despite this growth, data on CoCs in hair care and other PCPs available in the market remain limited. A 2023 survey of 302 salon workers in Kisumu City, Kenya, found that 88% of respondents recognized occupational exposure to cosmetics and PCPs as potential health risks (26).

The present study examines the ingredient label of selected hair relaxers and chemical straightening products reported by participants of a questionnaire-based study and available for purchase in Embu and Nakuru Counties, Kenya. The objective is to assess the presence of CoCs known to be associated with adverse health effects. Additionally, the study evaluates the pH levels of these relaxers to determine compliance with the Kenya Bureau of Standards (KEBS) regulations. KEBS is the governmental agency responsible for developing standards and ensuring quality control for various products, including nontherapeutic cosmetics such as hair relaxers in Kenya.

## 2 Materials and methods

### 2.1 Study design and data collection

This study received approval from the Institutional Review Board (IRB) of the Kenya Medical Research Institute (KEMRI/SERU/CTMDR/094/4138), the Columbia University IRB (AAU3921), and the National Commission for Science, Technology and Innovation (774442). The research was part of a broader cross-sectional, questionnaire-based study designed to assess the prevalence of hair product use, as well as attitudes and perceptions regarding potential health risks associated with chemical hair relaxers and other PCPs among women residing in Embu and Nakuru Counties, Kenya.

As previously described (27), research assistants approached potential participants in beauty shops, salons, and selected households, providing verbal information about the study. Eligible participants who consented to participate completed an interviewer-administered questionnaire, available in English and Kiswahili, which took approximately 20–30 min to complete. For participants aged 15–17 years, parental or guardian consent was obtained through an assent process for minors under age 18 years. The study focused on women aged 15–50 years, as existing literature indicates a high prevalence of relaxer use in this demographic (7, 8). Additionally, given the long latency period between chemical exposure and the onset of chronic diseases such as cancer, collecting data on product use at younger ages is particularly relevant. Recruitment and data collection were conducted from May 10 to July 28th, 2023, using a modified version of the PCP Use Questionnaire as previously described (27, 28).

**Figure 1:**
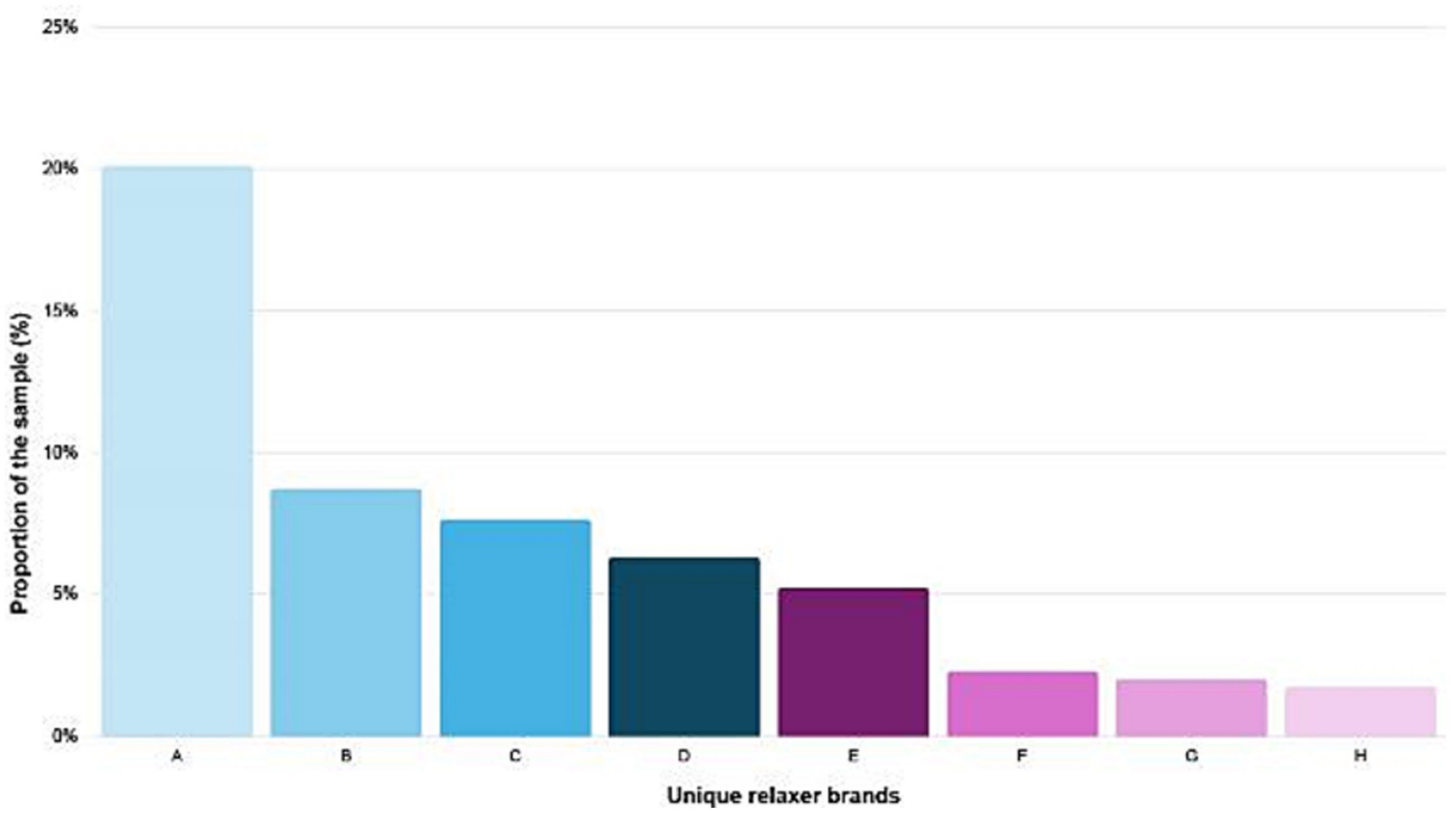
Distribution of the eight most frequently reported relaxer brands.

A total of 746 women from Embu and Nakuru Counties consented to participate and completed the questionnaire. The questionnaire collected sociodemographic data, information on recent (past 7–14 days) and historical (ever and past 12 months) use of hair products and other PCPs, and specific brand names of hair dyes and chemical relaxers. Based on participants’ questionnaire responses, 22 hair relaxers were selected for ingredients label review and pH analysis. Selection criteria included products from brands or manufacturers that were either reported by at least 10 participants as having been used at any time (the eight most frequently reported brands are presented in Figure 1; Supplementary Table 1) and/or those that were widely available in the local market across the two counties of interest. In addition to relaxers from eight most frequently reported brands, two additional brands were included based on product availability in Embu and Nakuru Counties.

For each selected relaxer, the printed ingredient labels were examined, and all listed components were documented. For relaxer kits containing multiple components (e.g., neutralizing shampoo, protective gel, activator cream or moisturizer), each component was analyzed as part of the overall product. Additional product label details, including relaxer strength, manufacturer, manufacturer’s location, and product use instructions, were also documented. The research team’s chemist (BNI) conducted an independent review to correct typographical and spelling errors and to remove duplicate ingredient listings.

CoCs were defined as any ingredient appearing on a product label that was also listed in either the Campaign for Safe Cosmetics’ (CSC) Red List, a program of Breast Cancer Prevention Partners in the United States [US] (10), or the European Union (EU) Prohibited/Restricted Substances Annex II and III, as outlined in Regulation 1223/2009/EC on Cosmetic Products, as amended by Regulation (EU) 2023/1490 (OJ L 183, 20 July 2023) (11, 12). The KS EAS 377–1: 2013 East African Standard on cosmetics and cosmetic products references the EU Annex II and III in regulating cosmetic safety. For comparative purposes, the CSC Red List was included in the evaluation, as it is widely used in the US to support advocacy efforts promoting safer cosmetics.

### 2.2 Chemicals of concern in hair relaxers and hair straightening products

To identify CoCs, the ingredients lists from each relaxer product were systematically cross-referenced against the CSC Red List and the EU Prohibited/Restricted Substances Annex II/ III. The frequency of CoCs appearing on product labels was also assessed.

The identified CoCs were categorized into three tiers per the CSC Red List (10), which includes chemicals used in cosmetics that have been associated with adverse health outcomes such as cancer, reproductive and developmental toxicity, and endocrine disruption. This classification is derived from authoritative lists compiled by regulatory and research organizations, including the International Agency for Research on Cancer (IARC), United States National Toxicology, EU Global Harmonized System (GHS) Codes and Pictograms, Association of Occupational and Environmental Clinics, European Union Fragrance allergen; Established Contact Allergens in Humans and European Chemical Agency Doc ED /77/2011. Additionally, some chemicals have been included in the CSC Red List based on evidence from peer-reviewed literature linking them with adverse health effects (29).

1. Tier 1 CoCs are classified as those chemicals that should be prohibited from use in PCPs or as fragrance ingredients by manufacturers and retailers;
2. Tier 2 CoCs are classified as emerging CoCs that should be avoided in products if possible; and
3. Tier 3 CoCs are classified as asthmagens, allergens or irritants whose presence in products should be disclosed as potential allergens.

### 2.3 Measurement of pH

Alkali-based hair relaxers, including those containing sodium, potassium, or calcium hydroxide, or guanidine carbonate, have pH values above neutral (>7) and are inherently corrosive. The pH levels of the 22 selected chemical hair relaxers were measured with a benchtop pH meter (HM-25G, JICS, Japan) equipped with a pH electrode and an integrated temperature probe, following methodology described by Sishi et al. (30). Briefly, five grams of each sample was extracted from the manufacturer’s packaging into a 50 mL beaker. The pH was measured according to the manufacturer’s instructions for the pH meter. To ensure accuracy, three independent pH readings were obtained for each sample over three consecutive days, and the mean value was recorded. The temperature of each product was documented at the time of pH measurement. For, no-lye relaxers (which contain calcium hydroxide and guanidine carbonate), the products were packaged with two components: a cream relaxer and cream activator. The pH of each component was measured individually before mixing. After combining the components using a wooden spatula, a follow-up pH measurement was conducted 24 h post-mixing to assess any changes in pH stability.

### 2.4 Statistical analysis

Statistical analysis was conducted using Analysis of Variance (ANOVA) in GraphPad Prism (version 8.2.0) to evaluate differences in mean pH values between sodium hydroxide (lye) relaxers versus calcium hydroxide/guanidine carbonate (no-lye) relaxers. Additionally, ANOVA was used to compare pH differences between sodium hydroxide relaxers labeled as “regular strength” versus “super strength.” A *p*-value <0.05 was considered statistically significant.

## 3 Results

### 3.1 Participant’s characteristics

The sociodemographic characteristics of the 746 study participants (372 from Embu County and 374 from Nakuru County) have been previously described by Llanos et al. (27). A summary is presented in Table 1. The mean age of participants was 30.4 ± 8.1 years. Regarding educational attainment, 19.0% had less than a high school education, while 80.9% had completed at least high school. In terms of marital status, 46.6% were married, and 46.1% had never married. Nearly half of the participants (48.8%) were employed in sales and service occupations, with a substantial proportion (40.5%) working as cosmetologists. More than half (57.4%) reporting a monthly income of less than Ksh 10,000. With respect to chemical relaxer use, 59.4% reported ever using relaxers, while 35.7% reported current use (within the past year). The majority (57.6%) indicated that they first used relaxers at age 20 years or older. Most participants (71.6%) typically had relaxers applied at a salon (71.6%) rather than self-administering them (12.2%). Additionally, 28.4% of participants reported using two or more different relaxer brands, and 45.4% did not know or could not recall whether the relaxers they typically used contained lye or not.

**Table 1:** Sociodemographic and relaxer use characteristics of study participants in Embu and Nakuru Counties, Kenya, *N* = 746.

Sociodemographic characteristics	n (%)
County of residence	
Embu	372 (49.9)
Nakuru	374 (50.1)
Age (years), mean ± SD	30.4 ± 8.1
Marital status	
Currently married	348 (46.6)
Formerly married	52 (7.0)
Never married	344 (46.1)
Education	
Less than high school certificate	142 (19.0)
High school certificate	283 (37.9)
Some college but no degree	253 (33.9)
Bachelor’s degree and above	67 (9.0)
Occupation field	
Business and administration	155 (20.8)
Education, government, and law enforcement	34 (4.6)
Farming and agriculture	60 (8.0)
Sales and service	364 (48.8)
Science, technology, and engineering	17 (2.3)
Student	61 (8.2)
Unemployed or casual work	49 (6.6)
Household income (Ksh per month)	
<10,000	427 (57.2)
10,000–50,000	279 (37.4)
>50,000	12 (1.6)
Chemical relaxer use characteristics	
Use of relaxers for ≥1 year (ever use)	
No	293 (39.3)
Yes	443 (59.4)
Use of relaxers in the past year (current use)	
No	449 (60.2)
Yes	266 (35.7)
Age relaxer use began (years)	
≤12	20 (4.5)
13–19	150 (33.9)
≥20	255 (57.6)
Typical relaxer application	
At-home	54 (12.2)
Salon	317 (71.6)
Both	56 (12.6)
Use of no-lye or lye relaxer products	
No-lye	71 (16.0)
Lye	96 (21.7)
Both	59 (13.3)
Do not know/cannot remember	201 (45.4)
Number of different relaxer brands used	
1	194 (43.8)
≥2	126 (28.4)

### 3.2 Classification of hair relaxers/straighteners

A total of 22 packaged hair relaxers and hair straightening products were purchased from beauty shops and supermarkets in Embu (*n* = 8) and Nakuru (*n* = 14) Counties. These products represented 10 different brands with nearly half of the brands being used by ≥40 study participants. The characteristics of the selected products are summarized in Table 2. Eight products (36.4%) were labeled normal/regular strength, 11 (50.0%) as super/extra strength, one (4.6%) as mild, and two (9.1%) as blow-out hair straighteners, which are designed to smooth and reduce frizz without chemically flattening the hair cuticle or altering the hair’s natural curl pattern. Regarding formulation, 15 products (68.2%) were lye-based relaxers containing sodium hydroxide, while seven were no-lye relaxers, including: two (9.1%) containing calcium hydroxide and five (22.7%) containing guanidine carbonate. All no-lye relaxers and two lye-based relaxers were packaged as kits, which included additional components such as neutralizing shampoo, protective gel, activator cream or moisturizer. In terms of manufacturing origin, 12 products were locally produced in Kenya, while 10 were imported.

**Table 2:** Characteristics of the 22 hair relaxer products evaluated.

Product code	Strength	Alkaline agent	Country of manufacture	Type of packaging^a^
EM1	Regular	Sodium hydroxide	Kenya	Kit
EM2	Blow-out	Sodium hydroxide	Kenya	Jar
EM3	Regular	Sodium hydroxide	Kenya	Jar
EM4	Super	Sodium hydroxide	Kenya	Jar
EM5	Super	Sodium hydroxide	Kenya	Kit
EM6	Super	Sodium hydroxide	Kenya	Jar
EM7	Mild	Sodium hydroxide	Uganda	Jar
EM8	Normal	Guanidine carbonate	South Africa	Kit
NK1	Regular	Sodium hydroxide	Kenya	Jar
NK2	Super	Sodium hydroxide	Kenya	Jar
NK3	Super	Sodium hydroxide	South Africa	Jar
NK4	Blow-out	Sodium hydroxide	Kenya	Jar
NK5	Regular	Sodium hydroxide	Uganda	Jar
NK6	Super	Sodium hydroxide	Kenya	Jar
NK7	Super	Guanidine carbonate	South Africa	Kit
NK8	Extra strength	Calcium hydroxide	South Africa	Kit
NK9	Regular	Guanidine carbonate	South Africa	Kit
NK10	Normal	Calcium hydroxide	Egypt	Kit
NK11	Super	Guanidine carbonate	Kenya	Kit
NK12	Regular	Guanidine carbonate	Kenya	Kit
NK13	Super	Sodium hydroxide	Uganda	Jar
NK14	Super	Sodium hydroxide	USA	Jar

a ‘Kit’ corresponds to relaxer products packaged in a box that contained one or more of the following: neutralizing shampoo, protective gel, and activator cream or moisturizer. ‘Jar’ corresponds to relaxer products that included a relaxer cream only.

### 3.3 Labelling requirements

**Figure 2:**
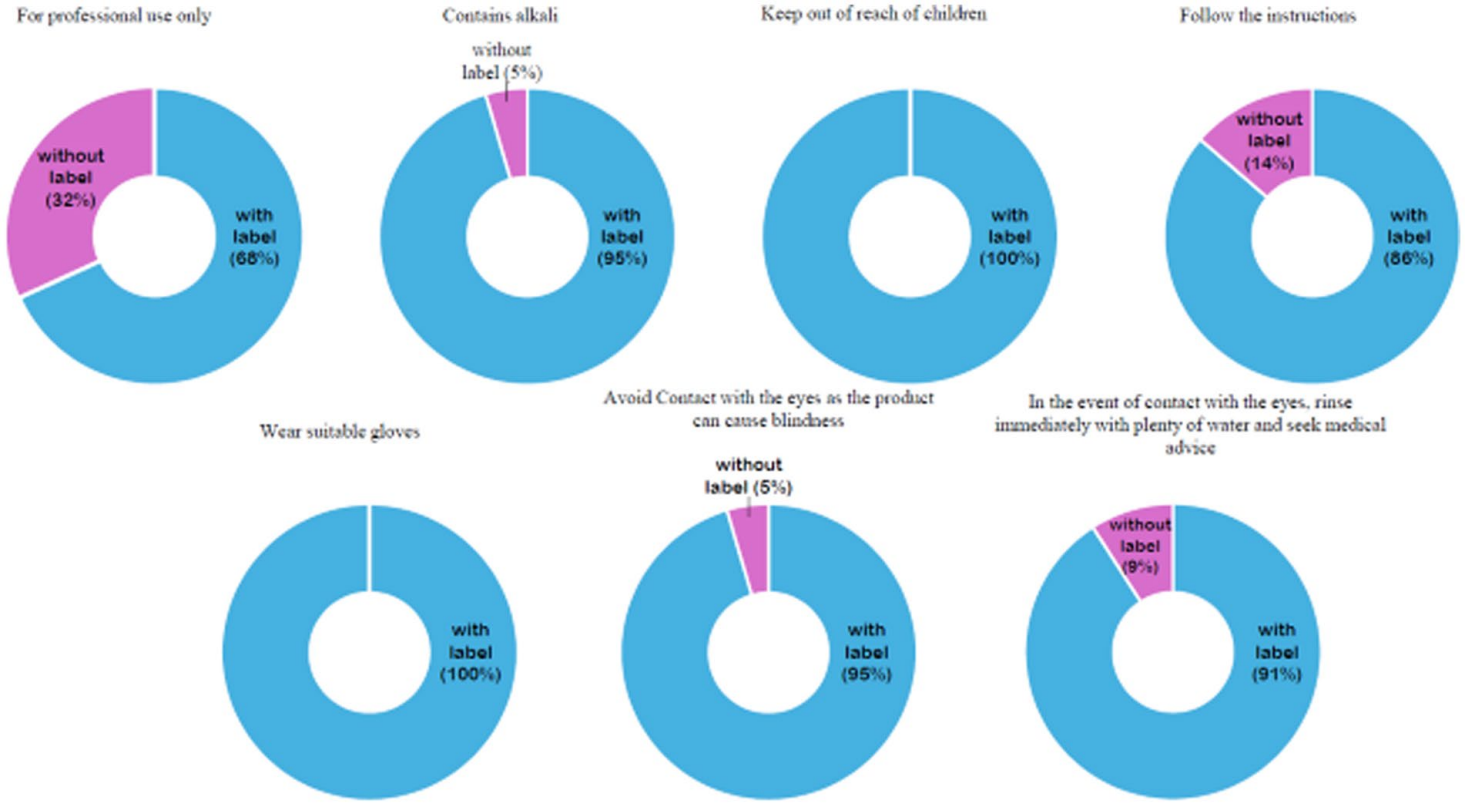
Distribution of warning labels found on chemical relaxer products.

All relaxer product labels were printed in English, in accordance with KEBS requirements (KS EAS 338:2013). Additionally, product labels were assessed for compliance with precautionary and warning statements mandated for lye and no-lye relaxers. Most products included the required precautionary and warning statements as shown in Figure 2. However, seven products (31.8%) did not include the “for professional use only” warning on the packaging, despite being a KEBS labeling requirement (KS EAS 338:2013).

### 3.4 Chemicals of concern listed in products’ ingredients lists

A total of 27 CoCs, as identified by the CSC Red List or the EU’s Annex II and III of Regulation (EC) No.1223/2009 on cosmetic products, were listed on the labels of the 22 analyzed hair relaxer products (Table 3). Three products from the same manufacturer listed titanium oxide and silica as ingredients. However, these substances were excluded from the final CoC list, as their classification as CoCs is based on the risk associated with inhalation of airborne particles, and none of the analyzed products were in powder form. Among the remaining 26 CoCs, classifications were distributed across the three tiers described by CSC’s Red List: six were Tier 1 CoCs, four were Tier 2 CoCs, and seven were Tier 3 CoCs. Notably, 10 CoCs (38.5%) were listed in both Tier 2 and Tier 3, indicating that, in addition to being emerging CoCs, they are also classified as asthmagens, allergens, or irritants (10). Furthermore, four ingredients were identified as prohibited substances, including phenolphthalein, which is banned from cosmetics products in the EU but was not included in the CSC Red List. Additionally, 15 substances were classified as restricted in the EU (31). Table 4 summarizes CoCs identified on relaxer product labels, their frequency, and typical uses. Nearly half (48.2%) of the identified CoCs were fragrances, with d-limonene/limonene and linalool appearing on labels of nine relaxer products.

**Table 3:** Chemicals of concern identified on ingredients lists of evaluated hair relaxer products.

Ingredient name	CAS number	CoC evidence list			Health concerns			
		Campaign for safe cosmetics red list (USA)	Annex II (EU)^a^	Annex III (EU)^b^	Cancer	Endocrine disruption	Reproductive developmental toxicity	Asthmagens, allergens, and irritants
Acrylic acid; carbomer	79–10-7	Tier 2 and 3						2a, 3a
*Aloe barbadensis* (leaf extract/ *aloe vera*)	85,507–69-3	Tier 2and 3			1a			2a
Benzyl alcohol	100–51-6	Tier 3		□				2a,5
Benzyl benzoate	120–51-4	Tier 2 and 3		□				5
Benzyl salicylate	118–58-1	Tier 1		□		6		2a, 5
Butylated hydroxytoluene (BHT)	128–37-0	Tier 1			2b	4	2c	2a
Calcium hydroxide	1,305-62-0	Tier 2 and 3		□				2a
Cinnamyl alcohol	104–54-1	Tier 3		□				2a, 5
Citral	5,392-40-5	Tier 2 and 3		□				2a, 5
Citronellol	106–22-9	Tier 2 and 3		□				2a, 5
CoCamide diethanolamine (CoCamide DEA)	68,603–42-9	Tier 1			1a			
Coumarin	91–64-5	Tier 2 and 3		□				2a, 5
D-limonene/limonene	5,989-27-5; 138–86-3	Tier 2 and 3		□				2a, 5
Formaldehyde	50–00-0	Tier 1	□		1b			2a, 3b
Geraniol	106–24-1	Tier 3		□				2a, 5
Hexyl cinnamal	101–86-0	Tier 3		□				2a, 5
Hydroxycitronellal	107–75-5	Tier 2 and 3		□				2a, 5
Isoeugenol	97–54-1	Tier 1		□	2b			2a, 5
Lilial	80–54-6	Tier 1	□				2d	2a, 5
Linalool	78–70-6	Tier 3		□				2a, 5
Mineral oils	8,012-95-1, 8,042-47-5, and others related to petroleum	Tier 3						2a
Petrolatum	8,009-03-8	Tier 2	□				2e	
Phenolphthalein	77–09-8	-	□		7			
Phenolsulfonphthalein (Phenol red)	143–74-8	Tier 2				6		
Phenoxyethanol	122–99-6	Tier 3						2a
Sodium hydroxide	1,310–73-2	Tier 2 and 3		□				2a
Tocopherol/Vitamin E	1,406-18-4	Tier 2				6		2a

a Prohibited Substances: Annex II, Regulation 1223/2009/EC on Cosmetic Products, as amended by Regulation (EU) 2023/1490, OJ L 183, 20 July 2023. This list contains substances which are banned from use in any cosmetic products marketed for sale or use in the European Union.

b Restricted Substances: Annex III, Regulation 1223/2009/EC on Cosmetic Products, as amended by Regulation (EU) 2023/1545, OJ L 188, 27 July 2023.

This list contains substances whose use in cosmetic products in the European Union is banned, except under certain conditions as indicated in Annex III. The list specifies the field of application or use, maximum allowable concentration limits in finished products, and any additional limitations. 1. International Agency for Research on Cancer (IARC) Monographs. a. Possible human carcinogen. b. Known Human Carcinogen. 2. Global harmonized system (GHS) codes and pictograms. a Asthmagens, allergens, and irritants (GHS 314, 315, 318, 319). b Suspected of causing cancer (GHS315). c Suspected of damaging fertility or unborn child. d Suspected of damaging fertility. e Suspected of damaging unborn child. 3. Association of Occupational and Environmental Clinics (AOEC). a Suspected Asthmagen. b Generally Accepted Asthmagen. 4. ChemSec Substitute It Now! (SIN) List (https://pharosproject.net/hazard-lists/307 accessed on 28/02/20240). 5. EU Fragrance Allergen: Established Contact Allergen in Humans. 6. Association supported by peer-reviewed literature according to Campaign for safe cosmetics list. 7. European Chemicals Agency Doc ED/77/2011: Inclusion of substances of very high concern in the candidate list (carcinogenic).

**Table 4:** Frequency of each chemical of concern listed on product labels of the 22 evaluated hair relaxer products and the typical use of the chemical.

Chemical name	Products listing the ingredient on the package label n (%)	Typical use of the chemical
*Aloe barbadensis* (leaf extract/*aloe vera*)	4 (18.2)	Antioxidant
Benzyl alcohol	3 (13.6)	Fragrance
Benzyl benzoate	3 (13.6)	Fragrance/antimicrobial/solvent
Benzyl salicylate	4 (18.2)	Fragrance
BHT (Butylated hydroxytoluene)	2 (9.1)	Antioxidant
Calcium hydroxide	7 (31.8)	Buffering
Carbomer	2 (9.1)	Emulsion stabilizing /gel forming
Cinnamyl alcohol	1 (4.6)	Fragrance
Citral	4 (18.2)	Fragrance
Citronellol	3 (13.6)	Fragrance
CoCamide diethanolamine (CoCamide DEA)	2 (9.1)	Surfactant/emulsifier
Coumarin	4 (18.2)	Fragrance
D-limonene/limonene	9 (40.9)	Fragrance
Formaldehyde	2 (9.1)	Preservative
Geraniol	2 (9.1)	Fragrance
Hexyl cinnamal	5 (22.7)	Fragrance
Hydroxycitronellal	3 (13.6)	Fragrance
Isoeugenol	2 (9.1)	Fragrance
Lilial	1 (4.6)	Fragrance
Linalool	9 (40.9)	Fragrance
Paraffinum liquidum (mineral oil)	20 (90.9)	Antistatic/emollient/solvent
Petrolatum	21 (95.5)	Antistatic/emollient
Phenolphthalein	2 (9.1)	pH level indicator
Phenolsulfonphthalein (Phenol red)	7 (31.8)	pH indicator
Phenoxyethanol	7 (31.8)	Preservative
Sodium hydroxide	16 (72.7)	Denaturant/buffering
Tocopherol/Vitamin E	5 (22.7)	Antioxidant

### 3.5 pH analysis

The pH values of all 22 analyzed relaxers were found to be within the KEBS regulatory limits (11–13, KS EAS 338:2013). The recorded pH values ranged from 13.0 to 13.4 for hydroxide-based relaxers (sodium and calcium) and 11.2 to 13.4 for guanidine carbonate relaxers (Supplementary Table 2). Comparative analysis of pH values based on chemical composition revealed no statistically significant differences among relaxers containing the three active ingredients (*p* = 0.53). Similarly, no significant differences were observed in paired comparisons, including lye vs. no-lye relaxers [sodium hydroxide vs. guanidine carbonate (*p* = 0.63) and sodium hydroxide vs. calcium hydroxide (*p* = 0.96)]. For sodium hydroxide relaxers, there was no statistically significant difference in pH levels between those labeled as super strength and regular strength (*p* = 0.53).

## 4 Discussion

As part of our ongoing research program investigating the prevalence and patterns of hair product and PCP use among women in Kenya, and the potential health implications associated with exposure to potentially toxic chemicals, this study examined the ingredients lists of 22 packaged relaxer products available on the market in Embu and Nakuru Counties. These products were reported for use in a population-based study in these two counties. The majority of analyzed products (68.2%) were readily available for over-the-counter purchase, despite bearing the “for professional use only” warning, as required by KEBS standards (KS EAS 338:2013). Among the seven products that did not include this warning on their packaging, it remained unclear whether they were intended for general consumer use. This raises a regulatory concern, as general-use hair relaxers are required to contain no more than 2% sodium hydroxide (31).

A total of 27 CoCs were identified in the ingredients lists of the evaluated relaxer products. These substances have been associated with various health effects, including cancer, endocrine disruption, reproductive and developmental toxicity, asthma, allergic reactions, and skin or eye irritation (10). Notably, CoCs were present across all 22 relaxer products, including within the neutralizing shampoos, protective gels, activator creams and moisturizers included in kit-packaged relaxers.

### 4.1 Chemicals of concern based on the CSC red list

Of the 27 CoCs identified, 26 were listed on the CSC Red List. CoCs categorized as Tier 1 (do not use CoCs for everyone) included fragrances, benzyl salicylate, isoeugenol, lilial, butylated hydroxytoluene (BHT, an antioxidant), cocamide diethanolamine (a surfactant/emulsifier), and formaldehyde (a preservative and known carcinogen). Formaldehyde, classified as a carcinogen, irritant, and an asthmagens, along BHT which is recognized as a possible carcinogen, irritant, and associated with reproduction and development harm and endocrine disruption are among the top 20 toxic chemicals to avoid in cosmetics as they have been linked to multiple adverse health effects (32). Interestingly*, Aloe barbadensis* leaf extract (*Aloe vera*), a natural product ingredient generally recognized as safe, is classified as an emerging CoC (Tier 2) and as a skin and eye irritant (Tier 3) according to the CSC Red List (10). Animal studies have demonstrated carcinogenic activity of *Aloe vera* whole-leaf extract, leading to its classification as a possible human carcinogen (Group 2B) by the IARC (33–35).

A total of 14 relaxer labels (63.6%) listed ‘fragrance’ and/or ‘parfum’ as ingredients without specifying the chemicals present. These terms typically refer to complex mixtures of undisclosed chemicals added to products to create a pleasant aroma or to mask the odor of strong ingredients, such as those in hair relaxers (14). Fragrance, listed among top 20 chemicals to avoid by the CSC, may contain allergens and EDCs, including phthalates and parabens, along with other compounds that pose potential health risks.

A recent US-based study evaluating 41 chemical hair relaxers for allergen content reported that fragrance was a common ingredient, appearing on more than half of the product labels (25/41, 61%) (36). In Kenya, the KEBS standard for cosmetic labeling (KS EAS 346:2022) requires that the term ‘perfume’ or ‘flavour’ be listed on product labels; however, there are no regulations mandating disclosure of the chemical constituents within these formulations. Notably, all relaxer product labels examined in this study lacked information on the potential adverse health effects of their listed ingredients, including perfume and/or fragrances. Similar findings were reported by Klaschka and Rother, who highlighted the limited availability of product label information and emphasized the need for greater consumer awareness regarding the potential health risks associated with PCPs (37).

### 4.2 Prohibited substances in the EU

The KEBS regulation (KS EAS 377–1: 2013) mandates that “*cosmetic products shall not contain any of the prohibited substances listed in Annex II of Regulation (EC) No.1223/2009 on cosmetic products of the European Parliament and of the Council”* as amended (11, 31). Among the 27 listed CoCs identified in this study, four (14.8%)—formaldehyde, lilial, petrolatum, and phenolphthalein—are classified as prohibited substances under EU cosmetic regulations (11, 31). Although petrolatum is widely used in cosmetics, its safety depends on proper refinement. According to Annex II (Prohibited Substances) of EU cosmetic regulations, petrolatum may only be used in cosmetic formulations if its full refining history is known and it can be demonstrated that the source material is not carcinogenic (11). In this study, 21 of the 22 hair relaxers/straighteners listed petrolatum as a major component, in accordance with cosmetic ingredient labeling standards, which require ingredients to be listed in descending order by volume (30, 37). Sishi et al. identified petrolatum as a primary component in 121 chemical hair relaxers marketed in South Africa (30). Additionally, a 2011 study observed an increase in mineral oil saturated hydrocarbons (MOSH) in human adipose tissue, suggested that cosmetics may be a relevant source of exposure (38).

Formaldehyde, commonly used as a preservative in cosmetics, is a documented contact allergen and has been classified as carcinogenic to humans (Group 1) by the IARC (39–41). It has also been linked to adverse health outcomes that disproportionately affect Black women, including maternal health complications and pregnancy-related risks (42). Experimental studies have demonstrated its carcinogenic potential in animal models, including work by Soffritti et al., which provided evidence of formaldehyde-induced carcinogenesis in rats (43). Additionally, epidemiologic studies suggest a probable association between formaldehyde exposure and leukemia in humans (44–46).

Phenolphthalein is classified as a possible carcinogen according to the European Chemical Agency (ECHA) and is included in the Substances of Very High Concern candidate list (47). Experimental studies have demonstrated its carcinogenic potential in animal models (48, 49). In this study, both formaldehyde and phenolphthalein were listed as ingredients in two neutralizing shampoos included in relaxer kits from the same manufacturer, but marketed in different strengths.

Lilial (butylphenyl methylpropional), a fragrance compound, was listed as an ingredient in a relaxer cream evaluated in this study. Lilial is a known contact allergen and has been associated with reproductive toxicity, including potential fertility impairment and skin sensitization risks (50, 51). The European Commission’s Scientific Committee on Consumer Safety has reported evidence of adverse effects on male reproductive function in animal studies following lilial exposure (50). As a result, lilial has been classified as a prohibited substance in all cosmetic products in the EU since March 2022 (11).

### 4.3 Restricted substances in the EU

Among the 27 CoCs identified in this study, 15 (55.6%) were classified as regulated but not prohibited substances under Annex III of Regulation (EC) No. 1223/2009. This regulation stipulates that cosmetic products may contain these substances only under specific restrictions (12). Notably, 13 of these 15 restricted substances (excluding sodium hydroxide and calcium hydroxide) were fragrance chemicals (52). The EU Scientific Committee on Consumer Safety (2012) has identified several fragrance CoCs due to their frequent association with contact allergies in humans, including benzyl alcohol, benzyl salicylate, cinnamyl alcohol, citral, coumarin, geraniol, hydroxycitronellal, and isoeugenol (52). Additional fragrance chemicals documented in the analyzed relaxer products included d-limonene, limonene, linalool, hexyl cinnamal, benzyl benzoate, and citronellol. All 22 relaxer products contained more than one fragrance chemical, indicating that users of these products are potentially exposed to multiple allergens and complex chemical mixtures. Animal studies have demonstrated that exposure to allergen mixtures may enhance both the induction and elicitation of contact allergies, as reported by Bonefeld et al. (53). According to Annex III of Regulation (EC) No. 1223/2009, fragrances must be disclosed on product labels if present above 0.001% in “leave-in” products or above 0.01% in “rinse-off” products (6). In this study, linalool and limonene/d-limonene, classified as skin and eye irritants, were the most frequently identified fragrance chemicals. These findings are consistent with prior research by Panico et al. in Italy and Buckley in the United Kingdom, both of whom reported limonene and linalool as the most commonly listed fragrance chemicals in cosmetic and household product labels (54, 55).

More than two-thirds (68.2%) of the hair relaxers analyzed were lye-based, containing sodium hydroxide as the active ingredient. Under EU harmonized classification and labeling policies, sodium hydroxide is classified as a corrosive agent that causes severe skin burns and eye damage (56). Hence, it is a restricted substance for cosmetic formulations, with a maximum allowable concentration of 4.5% in professional use products (12). The CSC Red List classifies sodium hydroxide as both a Tier 2 and Tier 3 CoC, labeling it as an emerging CoC and a potential skin and eye irritant (32). However, the Expert Panel for Cosmetic Ingredient Safety has concluded that hydroxides, including sodium hydroxide, are safe in hair straighteners when applied under manufacturer recommended conditions with minimal skin contact (57).

The high pH values observed in both lye and no-lye relaxers in this study align with the role of alkaline agents (sodium/calcium hydroxide and guanidine carbonate) in hair straightening formulations. These compounds raise the pH of relaxers, enabling the breakage of disulfide bonds in the hair cortex, which results in permanent straightening (9). Although the pH values of the 22 analyzed relaxers were within KEBS regulatory limits, their extreme alkalinity makes them inherently corrosive, increasing the risk of scalp burns and irritation, particularly when product use deviates from recommended guidelines. Evidence from Etemesi suggests that scalp burns are common among women using hair relaxers in Nakuru, Kenya, likely due to the highly alkaline nature of these products (8). Such burns may serve as potential entry points for CoCs into the body. Additionally, Geczik et al. reported that postmenopausal women who used lye-based relaxers and experienced a higher frequency of scalp burns exhibited altered estrogen metabolism, a factor that may contribute to adverse health outcomes (18). Although lye relaxers are generally considered to have a higher potential for scalp irritation compared to no-lye relaxers, the latter are often marketed as a safer option (58). However, in this study, no significant differences in the pH were observed between lye and no-lye relaxers or between different strengths of lye relaxers. These findings align with a study conducted by Sishi et al. in South Africa, which similarly found no significant differences in pH between calcium hydroxide and sodium hydroxide relaxers (*p* = 0.27) or between different strength formulations for sodium hydroxide relaxers (*p* = 0.90) (30).

### 4.4 Implications of chemical relaxer use among Kenyan women

Chemical hair relaxers remain a predominant method of hair straightening in Kenya. In this study, 35.7% of participants reported current use (within the past year) of chemical hair relaxers. However, there is limited data on the presence of CoCs in hair care products and other PCPs available on the Kenyan market, as well as their potential adverse health effects. Findings from this study indicate that selected hair relaxer products contain CoCs associated with a range of adverse health outcomes. Notably, formaldehyde a known carcinogen and a prohibited substance in all cosmetics under EU regulations was listed as an ingredient in two of the evaluated products. This underscores the need for further research to assess the potential health risks associated with chemical exposures from hair relaxers and other PCPs, including their possible role in cancer development and other conditions. Additionally, the data generated from this study can inform public health interventions and educational initiatives aimed at increasing awareness among consumers, cosmetologists, and salon workers regarding the potentially harmful chemicals present in hair products sold in Kenya. Such efforts could help promote safer product choices and better regulatory oversight in the Kenyan cosmetics industry.

### 4.5 Limitations of the study

This study is limited to a review of ingredients labels from 22 hair relaxers/chemical straighteners and measurement of their pH levels. The products analyzed were based on availability for purchase in two counties in Kenya and were identified through self-reported data from a questionnaire-based cross-sectional study, in which participants reported current and past use of specific relaxer brands and products. Given the reliance on self-reported product use, there is a possibility that some relaxer products not recalled at the time of the interview were omitted. Consequently, additional chemical relaxers used in these communities may not have been captured in this analysis. Furthermore, this study focused on one class of hair products, and while we documented the presence of 27 CoCs in relaxers, our findings do not represent the broader range of CoC exposures from other hair care products and PCPS. As such, the CoCs identified in relaxers sold in Embu and Nakuru Counties do not provide an exhaustive profile of potential chemical exposures that may contribute to adverse health outcomes in these communities. All analyzed products, including those labeled “for professional use only” were readily available for over-the-counter purchase in beauty shops and supermarkets. However, the study did not investigate whether regulatory bodies are actively enforcing restrictions on professional use only products. Despite these limitations, a key strength of this study is that it identified 27 CoCs that relaxer users in these two counties are likely exposed to. This provides an important initial assessment of potential chemical exposures in the population. Future research will include in-depth chemical analysis using mass spectrometry techniques to screen relaxer products, allowing a more comprehensive assessment of product composition and chemical concentrations. This will help to identify chemical ingredients that may not be disclosed on product labels, further enhancing our understanding of potentially harmful exposures among relaxer users. Additionally, expanding the scope of product classes analyzed will generate new insights into additional CoCs that may be present in PCPs beyond chemical relaxers.

## 5 Conclusion

All 22 hair relaxers analyzed in this study had at least one CoC listed on the product label. A total of 27 CoCs were documented, with 14 relaxer products (63.6%) listing undisclosed ingredients under the terms ‘fragrance’ and/or ‘parfum’. Additionally, four identified substances are classified as prohibited under EU Regulation (EC) No 1223/2009 on cosmetics, to which the KS EAS 377–1: 2013 East African Standard refers. Furthermore, 15 relaxers (68.2%) included the precautionary statement “for professional use only” on their labels; however, these products were readily available for over-the-counter purchase, raising concerns about regulatory enforcement and consumer safety awareness. To the best of our knowledge, this study is the first to document CoCs listed on the labels of selected relaxers sold in Embu and Nakuru Counties, contributing to the growing body of literature on harmful chemicals in cosmetics, particularly products disproportionately used among Black and African ancestry populations globally. Future laboratory-based analyses are necessary to detect the presence of undeclared CoCs in relaxers and to quantify the concentrations of both listed and unlisted chemicals. Additionally, this study highlights the urgent need for consumer education regarding potentially harmful chemicals in chemical relaxers marketed and sold in Kenya and their associated health risks.

## Data Availability

The original contributions presented in the study are included in the article/Supplementary material, further inquiries can be directed to the corresponding author.

## References

[1] Zota AR Shamasunder B. The environmental injustice of beauty: framing chemical exposures from beauty products as a health disparities concern. Am J Obstet Gynecol. (2017) 217:418.e1–6. doi: 10.1016/j.ajog.2017.07.020, PMID: 28822238 PMC5614862

[2] Collins HN Johnson PI Calderon NM Clark PY Gillis AD Le AM. Differences in personal care product use by race/ethnicity among women in California: implications for chemical exposures. J Expo Sci Environ Epidemiol. (2023) 33:292–300. doi: 10.1038/s41370-021-00404-7, PMID: 34952926 PMC10005944

[3] Llanos AA Rabkin A Bandera EV Zirpoli G Gonzalez BD Xing CY. Hair product use and breast cancer risk among African American and White women. Carcinogenesis. (2017) 38:883–92. doi: 10.1093/carcin/bgx060, PMID: 28605409 PMC5862263

[4] Brinton LA Figueroa JD Ansong D Nyarko KM Wiafe S Yarney J. Skin lighteners and hair relaxers as risk factors for breast cancer: results from the Ghana breast health study. Carcinogenesis. (2018) 39:571–9. doi: 10.1093/carcin/bgy002, PMID: 29324997 PMC6248529

[5] James-Todd T Senie R Terry MB. Racial/ethnic differences in hormonally-active hair product use: a plausible risk factor for health disparities. J Immigr Minor Health. (2012) 14:506–11. doi: 10.1007/s10903-011-9482-5, PMID: 21626298

[6] James-Todd T Terry MB Rich-Edwards J Deierlein A Senie R. Childhood hair product use and earlier age at menarche in a racially diverse study population: a pilot study. Ann Epidemiol. (2011) 21:461–5. doi: 10.1016/j.annepidem.2011.01.009, PMID: 21421329 PMC4116338

[7] Khumalo NP Jessop S Gumedze F Ehrlich R. Determinants of marginal traction alopecia in African girls and women. J Am Acad Dermatol. (2008) 59:432–8. doi: 10.1016/j.jaad.2008.05.036, PMID: 18694677

[8] Etemesi BA. Impact of hair relaxers in women in Nakuru. Kenya Int J Dermatol. (2007) 46:23–5. doi: 10.1111/j.1365-4632.2007.03458.x, PMID: 17919201

[9] Harrison S Sinclair R. Hair colouring, permanent styling and hair structure. J of Cosmetic Dermatol. (2003) 2:180–5. doi: 10.1111/j.1473-2130.2004.00064.x, PMID: 17163926

[10] CSC. Red list. Safe Cosmetics (2024). Available online at: https://www.safecosmetics.org/red-list/ [Accessed May 17, 2024]

[11] ECHA. Cosmetics-prohibited-subs - ECHA. (2023). Available online at: https://echa.europa.eu/cosmetics-prohibited-substances?p_p_id=eucleflegislationlist_WAR_euclefportlet&p_p_lifecycle=0 [Accessed May 17, 2024]

[12] ECHA. Cosmetics-restricted-subs - ECHA. (2023). Available online at: https://echa.europa.eu/cosmetics-restricted-substances?p_p_id=eucleflegislationlist_WAR_euclefportlet&p_p_lifecycle=0 [Accessed May 17, 2024]

[13] Helm JS Nishioka M Brody JG Rudel RA Dodson RE. Measurement of endocrine disrupting and asthma-associated chemicals in hair products used by black women. Environ Res. (2018) 165:448–58. doi: 10.1016/j.envres.2018.03.030, PMID: 29705122

[14] Johnson PI Favela K Jarin J Le AM Clark PY Fu L. Chemicals of concern in personal care products used by women of color in three communities of California. J Expo Sci Environ Epidemiol. (2022) 32:864–76. doi: 10.1038/s41370-022-00485-y, PMID: 36323919 PMC9628299

[15] Eberle CE Sandler DP Taylor KW White AJ. Hair dye and chemical straightener use and breast cancer risk in a large US population of black and white women. Int J Cancer. (2019) 147:383–91. doi: 10.1002/ijc.32738 31797377 PMC7246134

[16] White AJ Gregoire AM Taylor KW Eberle C Gaston S O’Brien KM. Adolescent use of hair dyes, straighteners and perms in relation to breast cancer risk. Int J Cancer. (2021) 148:2255–63. doi: 10.1002/ijc.33413, PMID: 33252833 PMC7969396

[17] McDonald JA Tehranifar P Flom JD Terry MB James-Todd T. Hair product use, age at menarche and mammographic breast density in multiethnic urban women. Environ Health. (2018) 17:1. doi: 10.1186/s12940-017-0345-y, PMID: 29301538 PMC5753455

[18] Geczik AM Falk RT Xu X Wiafe-Addai B Yarney J Awuah B. Relation of circulating estrogens with hair relaxer and skin lightener use among postmenopausal women in Ghana. J Expo Sci Environ Epidemiol. (2023) 33:301–10. doi: 10.1038/s41370-021-00407-4, PMID: 34992224 PMC9256865

[19] Wise LA Wang TR Ncube CN Lovett SM Abrams J Boynton-Jarrett R. Use of chemical hair straighteners and fecundability in a north American preconception cohort. Am J Epidemiol. (2023) 192:1066–80. doi: 10.1093/aje/kwad079, PMID: 37005071 PMC10505421

[20] Wise LA Palmer JR Reich D Cozier YC Lynn R. Hair relaxer use and risk of uterine leiomyomata in African-American women - PMC. (2011). Available online at: https://www.ncbi.nlm.nih.gov/pmc/articles/PMC3282879/ [Accessed March 14, 2024] 10.1093/aje/kwr351 PMC3282879 22234483

[21] Bertrand KA Delp L Coogan PF Cozier YC Lenzy YM Rosenberg L. Hair relaxer use and risk of uterine cancer in the black Women’s health study. Environ Res. (2023) 239:117228. doi: 10.1016/j.envres.2023.117228, PMID: 37821068 PMC10842360

[22] Chang C-J O’Brien KM Keil AP Gaston SA Jackson CL Sandler DP. Use of straighteners and other hair products and incident uterine Cancer. J Natl Cancer Inst. (2022) 114:1636–45. doi: 10.1093/jnci/djac165, PMID: 36245087 PMC9949582

[23] White AJ Sandler DP Gaston SA Jackson CL O’Brien KM. Use of hair products in relation to ovarian cancer risk. Carcinogenesis. (2021) 42:1189–95. doi: 10.1093/carcin/bgab056, PMID: 34173819 PMC8561257

[24] Uber M Morgan MA Schneider MC Gomes IR Imoto RR Carvalho VO. Frequency of perfume in 398 children’s cosmetics. J Pediatr. (2024) 100:263–6. doi: 10.1016/j.jped.2023.11.002, PMID: 38012955 PMC11065650

[25] The Star. An overview of the Kenyan beauty industry: trends and opportunities. Star (2023). Available online at: https://www.the-star.co.ke/news/2023-01-12-an-overview-of-the-kenyan-beauty-industry-trends-and-opportunities/ [Accessed September 25, 2024]

[26] Onyango PO. The cost of beauty: perspectives of salon workers in Kisumu City, Kenya. PLOS Glob Public Health. (2023) 3:e0002503. doi: 10.1371/journal.pgph.0002503, PMID: 37930951 PMC10627437

[27] Llanos AA Ashrafi A Olisa T Rockson A Schaefer A McDonald JA. Hair dye and relaxer use among cisgender women in Embu and Nakuru counties, Kenya: associations with perceived risk of breast Cancer and other health effects. Int J Environ Res Public Health. (2024) 21:846. doi: 10.3390/ijerph21070846, PMID: 39063423 PMC11277196

[28] Llanos AA Rockson A Getz K Greenberg P Portillo E McDonald JA. Assessment of personal care product use and perceptions of use in a sample of US adults affiliated with a university in the northeast. Environ Res. (2023) 236:116719. doi: 10.1016/j.envres.2023.116719, PMID: 37481059 PMC10592243

[29] BCPPs, CSC. Chemicals of concern. (2018). Available online at: https://www.safecosmetics.org/red-list/ [Accessed March 1, 2023]

[30] Sishi VNB Van Wyk JC Khumalo NP. The pH of lye and no-lye hair relaxers, including those advertised for children, is at levels that are corrosive to the skin. SAMJ: South African Med J. (2019) 109:941–6. doi: 10.7196/SAMJ.2019.v109i12.14010, PMID: 31865956

[31] UNION P. Regulation (EC) No 1223/2009 of the european parliament and of the council. Off J Eur Union L. (2009). 342:59.

[32] CSC. Top toxic ingredients in cosmetics Safe Cosmetics (2024). Available online at: https://www.safecosmetics.org/toxic-ingredients/ [Accessed March 25, 2024]

[33] Boudreau MD Mellick PW Olson GR Felton RP Thorn BT Beland FA. Clear evidence of carcinogenic activity by a whole-leaf extract of Aloe barbadensis miller (aloe vera) in F344/N rats. Toxicol Sci. (2013) 131:26–39. doi: 10.1093/toxsci/kfs275, PMID: 22968693 PMC3537128

[34] Boudreau MD Beland FA Nichols JA Pogribna M. Toxicology and carcinogenesis studies of a nondecolorized [corrected] whole leaf extract of Aloe barbadensis miller (Aloe vera) in F344/N rats and B6C3F1 mice (drinking water study). Natl Toxicol Program Tech Rep Ser. (2013) 577:1–266. PMID: 24042237

[35] Guo X Mei N. *Aloe vera*: a review of toxicity and adverse clinical effects. J Environ Sci Health C Environ Carcinog Ecotoxicol Rev. (2016) 34:77–96. doi: 10.1080/10590501.2016.1166826, PMID: 26986231 PMC6349368

[36] Okeke CAV Seltzer JA De Guzman CB Tran JH Okoye GA Byrd AS. Allergen content of popular chemical hair relaxers: a product analysis. Contact Derm. (2024) 91:139–45. doi: 10.1111/cod.14583, PMID: 38783163

[37] Klaschka U Rother H-A. ‘Read this and be safe!’ Comparison of regulatory processes for communicating risks of personal care products to European and south African consumers. Environ Sci Eur. (2013) 25:30. doi: 10.1186/2190-4715-25-30

[38] Concin N Hofstetter G Plattner B Tomovski C Fiselier K Gerritzen K. Evidence for cosmetics as a source of mineral oil contamination in women. J Women's Health. (2011) 20:1713–9. doi: 10.1089/jwh.2011.2829, PMID: 21970597

[39] Lundov MD Moesby L Zachariae C Johansen JD. Contamination versus preservation of cosmetics: a review on legislation, usage, infections, and contact allergy. Contact Derm. (2009) 60:70–8. doi: 10.1111/j.1600-0536.2008.01501.x, PMID: 19207376

[40] The International Agency for Research on Cancer. “A review of human carcinogens.” *Part F: Chemical agents and related occupations / IARC working group on the evaluation of carcinogenic risks to humans (2009: Lyon, France)*. (2009).

[41] Malinauskiene L Blaziene A Chomiciene A Isaksson M. Formaldehyde may be found in cosmetic products even when unlabelled. Open Med (Wars). (2015) 10:323–8. doi: 10.1515/med-2015-0047, PMID: 28352713 PMC5152996

[42] BCPP. TIER 1: "do not use “chemicals linked to health outcomes of greater concern to women.” (2022). Available online at: https://www.safecosmetics.org/wp-content/uploads/2022/08/BCPP-Red-List-for-Web_Tier-1_Black-Health-COC-_Key.pdf [Accessed September 19, 2024]

[43] Soffritti M Belpoggi F Lambertin L Lauriola M Padovani M Maltoni C. Results of long-term experimental studies on the carcinogenicity of formaldehyde and acetaldehyde in rats. Ann N Y Acad Sci. (2002) 982:87–105. doi: 10.1111/j.1749-6632.2002.tb04926.x 12562630

[44] Zhang L Tang X Rothman N Vermeulen R Ji Z Shen M. Occupational exposure to formaldehyde, hematotoxicity, and leukemia-specific chromosome changes in cultured myeloid progenitor cells. Cancer Epidemiol Biomarkers Prev. (2010) 19:80–8. doi: 10.1158/1055-9965.EPI-09-0762, PMID: 20056626 PMC2974570

[45] Hauptmann M Lubin JH Stewart PA Hayes RB Blair A. Mortality from Lymphohematopoietic malignancies among Workers in Formaldehyde Industries. JNCI J Natl Cancer Inst. (2003) 95:1615–23. doi: 10.1093/jnci/djg083, PMID: 14600094

[46] Pinkerton LE Hein MJ Stayner LT. Mortality among a cohort of garment workers exposed to formaldehyde: an update. Occup Environ Med. (2004) 61:193–200. doi: 10.1136/oem.2003.007476, PMID: 14985513 PMC1740723

[47] ECHA. Candidate list of substances of very high concern for authorisation - ECHA. (2011). Available online at: https://echa.europa.eu/candidate-list-table/-/dislist/details/0b0236e1807dc152 [Accessed March 19, 2024]

[48] Dunnick JK Hailey JR. Phenolphthalein exposure causes multiple carcinogenic effects in experimental model systems. Cancer Res. (1996) 56:4922–6. 8895745

[49] Stoll RE Blanchard KT Stoltz JH Majeska JB Furst S Lilly PD. Phenolphthalein and Bisacodyl: assessment of genotoxic and carcinogenic responses in heterozygous p53 (+/−) mice and Syrian Hamster embryo (SHE) assay. Toxicol Sci. (2006) 90:440–50. doi: 10.1093/toxsci/kfj081, PMID: 16373391

[50] Bernauer U Bodin L Celleno L Chaudhry Q Coenraads PJ. SCCS preliminary OPINION ON the safety of Butylphenyl methylpropional (p-BMHCA) in cosmetic products"-submission II, ref CCS/1591/17-preliminary version. (2017). Available online at: https://hal.science/hal-01669154/document [Accessed March 20, 2024]

[51] United Nations. GHS classification summary. GHS classification (Rev10, 2023) summary (2023). Available online at: https://pubchem.ncbi.nlm.nih.gov/ghs/ [Accessed March 8, 2024]

[52] SCCS. Opinion on fragrance allergens in cosmetic products. (2012). Available online at: https://ec.europa.eu/health/scientific_committees/consumer_safety/docs/sccs_o_102.pdf [Accessed February 29, 2024]

[53] Bonefeld CM Nielsen MM Rubin IMC Vennegaard MT Dabelsteen S Gimenéz-Arnau E. Enhanced sensitization and elicitation responses caused by mixtures of common fragrance allergens. Contact Derm. (2011) 65:336–42. doi: 10.1111/j.1600-0536.2011.01945.x, PMID: 21767274

[54] Panico A Serio F Bagordo F Grassi T Idolo A De Giorgi M. Skin safety and health prevention: an overview of chemicals in cosmetic products. J Prev Med Hyg. (2019) 60:E50. doi: 10.15167/2421-4248/jpmh2019.60.1.1080 31041411 PMC6477564

[55] Buckley DA. Fragrance ingredient labelling in products on sale in the UK. Br J Dermatol. (2007) 157:295–300. doi: 10.1111/j.1365-2133.2007.08018.x, PMID: 17573873

[56] ECHA. Substance information - sodium hydroxide. (2023). Available online at: https://echa.europa.eu/substance-information/-/substanceinfo/100.013.805 [Accessed March 7, 2024]

[57] Burnett CL Bergfeld WF Belsito DV Hill RA Klaassen CD Liebler DC. Safety assessment of inorganic hydroxides as used in cosmetics. Int J Toxicol. (2021) 40:16S–35S. doi: 10.1177/10915818211018381, PMID: 34514896

[58] Richardson V Agidi AT Eaddy ER Davis LS. Ten pearls every dermatologist should know about the appropriate use of relaxers. J of Cosmetic Dermatol. (2017) 16:9–11. doi: 10.1111/jocd.12262, PMID: 27472987

