# Supplementary material for: Chemicals of concern in select packaged hair relaxers available on the Kenyan market: an examination of ingredient labels and measurement of pH
Source: Front Public Health. 2025 Apr 16;13:1532113. doi: 10.3389/fpubh.2025.1532113 (PMC12042934; doi:10.3389/fpubh.2025.1532113)
Supplement: Supplementary file 1 [file Table_1.docx]

**Supplementary Table 1:** Brand name associated with the top eight relaxer product brands as reported by study participants

| **Brand Code*** | **Brand Name** |
| --- | --- |
| **A** | TCB Naturals |
| **B** | Movit |
| **C** | Dark and Lovely |
| **D** | Nice & Lovely |
| **E** | Miadi |
| **F** | Sofn’Free |
| **G** | Venus |
| **H** | Protective Mega Growth |

*These brand codes represent the unique relaxer brands as shown in Figure 1. The two additional relaxer products selected based on product availability in Embu and Nakuru Counties were from the brands Organic Root Stimulator (ORS) Olive Oil and Sta-Sof-Fro.
