# Supplementary material for: Chemicals of concern in select packaged hair relaxers available on the Kenyan market: an examination of ingredient labels and measurement of pH
Source: Front Public Health. 2025 Apr 16;13:1532113. doi: 10.3389/fpubh.2025.1532113 (PMC12042934; doi:10.3389/fpubh.2025.1532113)
Supplement: Supplementary file 2 [file Table_2.docx]

**Supplementary Table 2:** pH values reported for the 22 relaxer products evaluated

| **Product Code** | **pH Value** |
| --- | --- |
| EM1 | 13.29 |
| EM2 | 13.37 |
| EM3 | 13.27 |
| EM4 | 13.28 |
| EM5 | 13.26 |
| EM6 | 13.32 |
| EM7 | 13.22 |
| EM8 | 11.19 |
| NK1 | 13.13 |
| NK2 | 13.24 |
| NK3 | 13.13 |
| NK4 | 13.18 |
| NK5 | 13.14 |
| NK6 | 13.03 |
| NK7 | 13.35 |
| NK8 | 13.35 |
| NK9 | 13.23 |
| NK10 | 13.39 |
| NK11 | 13.23 |
| NK12 | 13.27 |
| NK13 | 13.14 |
| NK14 | 13.18 |
